# Case Report: Hip arthroplasty after fracture-related joint infection caused by extensively drug-resistant *Klebsiella pneumoniae*

**DOI:** 10.3389/fsurg.2024.1363298

**Published:** 2024-02-27

**Authors:** Maximilian Fischer, Lars Nonnenmacher, Johannes C. Reichert, Jürgen A. Bohnert, Evgeny A. Idelevich, Eyüp Doğan, Karsten Becker, Georgi I. Wassilew

**Affiliations:** ^1^ Center for Orthopaedics, Trauma Surgery and Rehabilitation Medicine, University Medicine Greifswald, Greifswald, Germany; ^2^ Friedrich Loeffler-Institute of Medical Microbiology, University Medicine Greifswald, Greifswald, Germany; ^3^ Institute of Medical Microbiology, University Hospital Münster, Münster, Germany

**Keywords:** acetabular fracture, hip arthroplasty, fracture-related infection, joint infection, musculoskeletal infection, multidrug-resistant pathogens, *Klebsiella pneumoniae*, cefiderocol

## Abstract

This case-report focuses on a 23-year-old soldier suffering from a fracture-related hip joint infection (FRI) due to extensively drug-resistant *Klebsiella pneumoniae* and *S. epidermidis*. The patient underwent multiple septic revision surgeries including the removal of remaining shrapnel accompanied by last-resort antimicrobial therapy with cefiderocol and colistin. Additionally, the surgeries included repeated tissue sampling for microbiological and histopathological analysis. An antibiotic-loaded cemented filler containing cefiderocol was used to improve local antimicrobial therapy. The biopsies prior to and during hip replacement surgery confirmed successful microbe eradication. Hip arthroplasty restored hip joint function and significantly improved patient's quality of life. The utilization of a trabecular metal shell and a meta-diaphyseally anchored cementless hip stem ensured secure implant fixation and early patient mobilisation. An adjusted biofilm active oral antimicrobial therapy after arthroplasty intervention was continued to prevent early periprosthetic joint infection. This case emphasizes the difficulties of managing FRI and multidrug-resistant pathogens. It contributes valuable insight into navigating complex orthopedic cases while ensuring successful hip arthroplasty outcomes. In conclusion, early interdisciplinary collaboration, appropriate antimicrobial therapy along with tailored surgical interventions are crucial for managing such complex cases successfully.

## 1 Introduction

Fracture-related infections (FRI) representing major complications in musculoskeletal surgery resulting in devasting patient burden (1). FRI appear in overall 5% of fractures, assuming over 1.8 mio FRI worldwide per year. In the pathogenesis of FRI Staph. aureus and Staph. epidermidis are the most frequent pathogens, although gram-negative bacteria are frequently identified in FRI related to pelvic and open fractures (2). Polymicrobial infections are common, occurring in up to one third of cases (3, 4).

Several risk factors for FRI has already been described, including patient individual factors, open fractures with severe soft tissue trauma and wound contamination with foreign material (5, 6).

Recently, armed conflicts have led to a surge in complex musculoskeletal injuries, often involving fracture contamination by foreign material, resulting in high proportions of severe secondary multidrug-resistant tissue infections (7, 8). Considering this situation, the combination with an extensive soft-tissue damage due to exploding bombs, grenades and mines creates an even higher therapeutic dilemma (9). In such situations, adequate treatment is crucial, including surgical debridement, foreign body removal as well as antimicrobial therapy to prevent mortality (10).

In case of extensive hip joint injuries caused by gun-shots, shrapnel's or explosions at the battlefields, total hip arthroplasty (THA) is often required to restore patients joint function (11, 12).

The rising prevalence of multidrug-resistant pathogens is one of the most pressing challenges in medicine of the 21st century (13). Even in orthopaedic surgery, the presence of multi-drug resistant septic arthritis poses a substantial therapeutic challenge (14). Consequently, the complication rates and the risk of periprosthetic joint infection are known to be significantly elevated in the light of previous septic arthritis. Besides intrinsic antibiotic resistance by certain bacteria, acquired resistance by mutation and horizontal gene transfer reducing the therapeutic options significantly (15). The Gram-negative enterobacterial species *Klebsiella pneumoniae* has gained infamy due to the rising number of severe infections and high mortality rates (16, 17). Their mechanism of resistance commonly involves the presence of extended-spectrum *β*-lactamases (ESBL) and the expression of carbapenemases (18, 19).

As a result, last-resort drug therapy is often needed in light of musculoskeletal and implant-associated infections, resulting in a high patient burden due to drug side effects and long-term hospitalization (20–22).

Consequently, this report seeks to elucidate the interplay between war-related injuries, pelvic FRI due to extensively drug-resistant *K. pneumonia*e, with particular attention to the unique challenges posed by the restricted options for antimicrobial drug therapy as well as surgical arthroplasty interventions. Additionally, we will explore the success and outcomes of hip arthroplasty in this context.

This case analysis offers a distinctive perspective on the intricate challenges and advancements in addressing septic hip arthritis and multidrug-resistant pathogens in orthopedics, emphasizing the context of constrained arthroplasty possibilities. By navigating this complex terrain, we aim to enhance the understanding and advance the approach to managing these complex orthopedic cases, particularly within the realm of war-induced injuries and their ramifications on the success of hip arthroplasty.

## 2 Case description

A 23-years old male soldier (BMI 22.2 kg/m^2^, no history of comorbidities) with a complex acetabular and hip joint fracture caused by a shrapnel war injury was admitted to our institution in July 2022. The shrapnel shuttered the left hip joint and caused a bladder injury consecutively. Additionally, the shrapnel exit resulted in an incomplete injury of the right femoral nerve.

Further gunshot wounds resulting in a left tibial plateau fracture as well as a left shuttered elbow and metatarsus fracture. These injuries were initially treated in the patient's home country.

**Figure 1:**
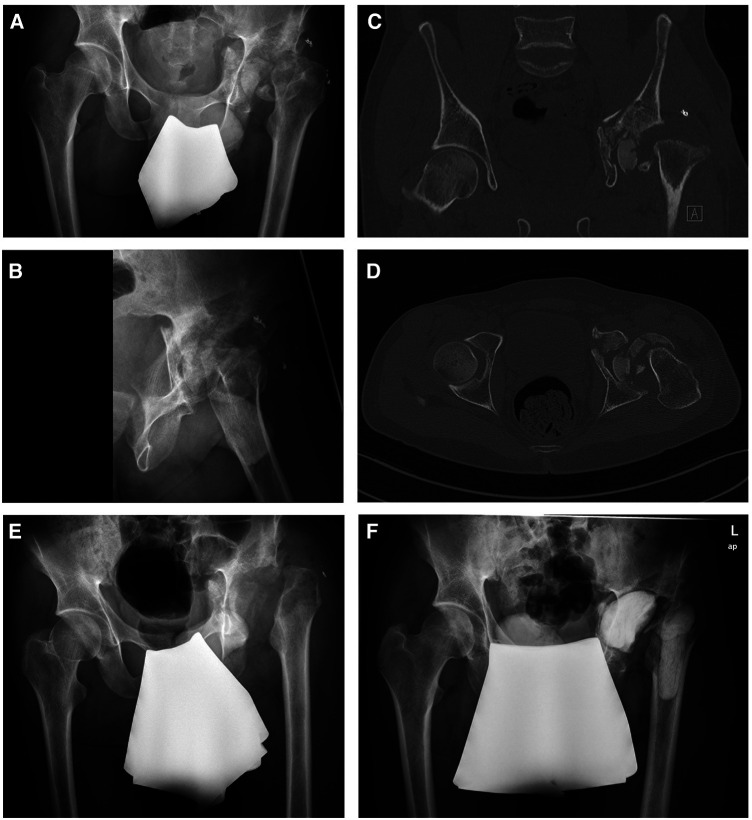
Preoperative x-rays (**A,B**) and CT scans showing a multi-fragmentary acetabular and femoral head fracture. After septic debridement, removal of remaining foreign bodies and removal of osseous fragments (**E**), an antibiotic-loaded cemented filler was implanted to the left acetabulum and proximal femur (**F**).

During the admission examination, the cardiopulmonary stable and responsive patient presented with non-irritating skin conditions observed over the left hip joint and the hip joint flexion was limited to 45°. Prior to the first surgical intervention, a comprehensive radiographic assessment, including x-ray and CT scans of the pelvis (Figure 1) and a hip joint aspiration (no bacterial growth) were performed. The CT scan revealed a multi-fragmentary acetabular and proximal femur fracture with pelvic dislocation of osseous fragments. No radiographic signs of bone consolidation were observed months after the initial trauma, raising suspicion of an infection. Therefore, the first surgical intervention consisted of septic debridement, removal of remaining foreign bodies followed by extensive lavage and a girdlestone procedure of the left hip with 5 intraoperative biopsies taken for subsequent microbiological analysis. The initial histopathological assessment from tissue samples taken during the first surgical procedure showed signs of an chronic septic infection.

**Figure 2:**
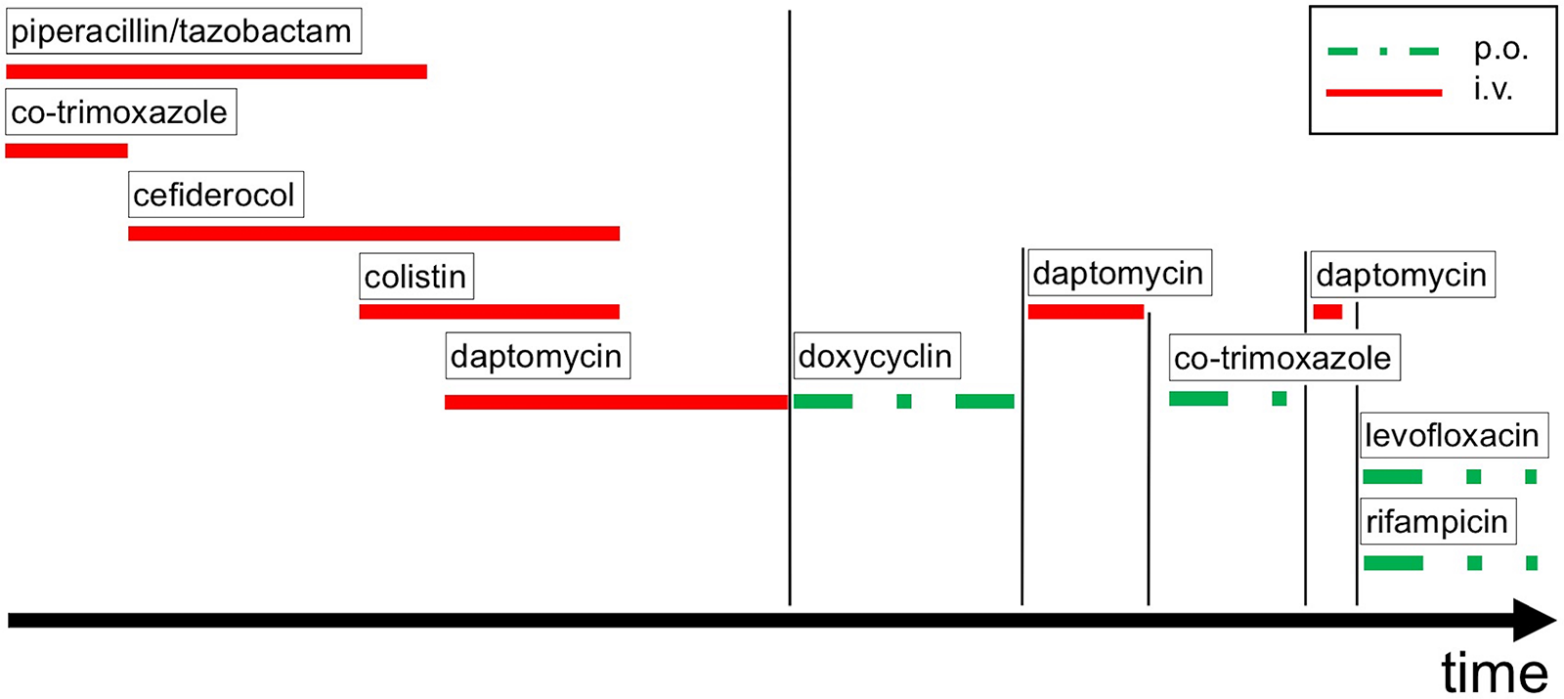
Antimicrobial drug therapy over time. p.o. oral application, i.v. intravenous application.

Antimicrobial therapy with piperacillin-tazobactam and co-trimoxazole was initiated after *K. pneumoniae* was cultivated from the removed tissue samples by mid-July 2022 (Figure 2). The microbiological examination revealed two carbapenem-resistant *K. pneumoniae* strains that harbored OXA-48/NDM and OXA-48 carbapenemases (as determined by LAMP (loop-mediated isothermal amplification), respectively. Accordingly, the antimicrobial therapy was changed to cefiderocol, a newly available siderophore cephalosporin (Figure 2). The OXA-48 carrying strain was found to exert susceptibility to ceftolozan-tazobactam and ceftazidime-avibactam, whereas the OXA-48/NDM carrying strain was resistant to these antibiotics. Both strains were resistant to ciprofloxacin but susceptible to colistin and co-trimoxazole in the initial antimicrobial susceptibility testing, hence the strains were categorized as multidrug-resistant (MDR).

While the OXA-48/NMD carrying *K. pneumoniae* strain remained detectable in the tissue samples until the end of September 2022, the more susceptible OXA-48 carrying strain disappeared from the tissue samples soon after the start of the cefiderocol therapy. Interestingly, while the more resistant OXA-48/NMD carrying strain initially displayed co-trimoxazole sensitivity, it had acquired resistance to this drug by early September 2022. Given that the strain was, in addition, resistant to all beta lactam / beta lactamase inhibitor combinations as well as aminoglycosides, fluoroquinolones, fosfomycin and tigecycline, and retained only susceptibility to cefiderocol and colistin, the strain was re-classified as extensively drug-resistant (XDR). Thus, colistin was added to the ongoing antimicrobial therapy (Figure 2).

Additionally, by mid-October 2023, a methicillin-resistant *S. epidermidis* was subsequently detected in all collected tissue samples, whereas the *K. pneumoniae* XDR strain could not be cultivated, anymore. Hence, daptomycin was added to the cefiderocol and colistin regimen (Figure 2).

**Figure 3:**
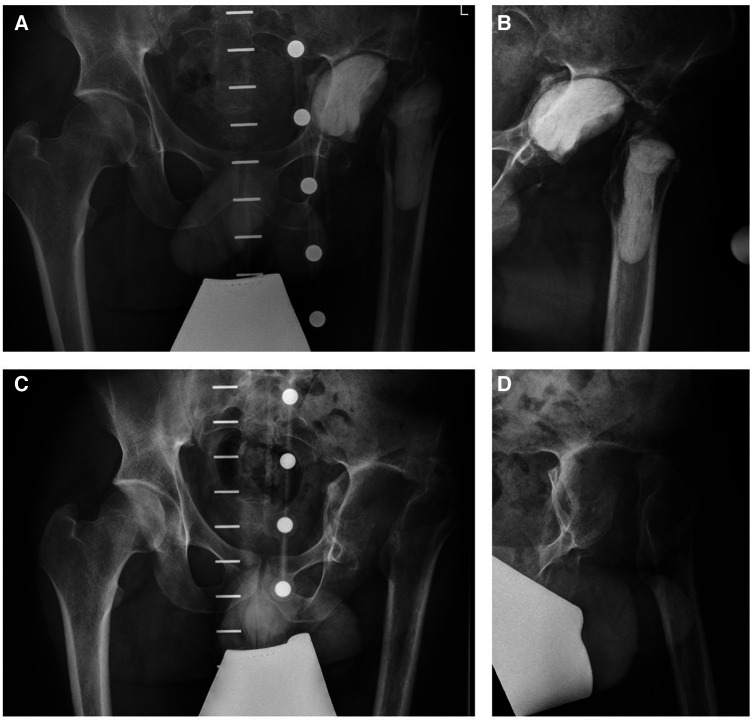
Pelvic x-rays (**A,B**) showing the customized cemented filler containing gentamicin, vancomycin and cefiderocol. Foreign body removal was performed before hip arthroplasty implantation (**C,D**).

In order to improve the local antimicrobial drug delivery, a drug-eluting customized cemented filler containing gentamicin, vancomycin and cefiderocol was implanted to the left acetabulum und proximal femur (Figures 3A,B). Consecutive blood chemistry analysis was performed three times a week to monitor the antimicrobial therapy as well as possible adverse side effects including potential hepatic and kidney toxicity.

A total of seven septic revision surgeries, including repeated tissue sampling for microbiological and histopathological analysis along with septic debridement, in combination with a local and systemic antimicrobial drug therapy using the last-resort drugs cefiderocol/ colistin and daptomycin were necessary to eradicate the XRD *K. pneumoniae* and to achieve sufficient wound healing. Additionally, wound conditioning was supported by superficial application of cold physical plasma (CPP).

In a multidisciplinary approach it was consented to allow soft tissue recovery over a period of three months and to continue an oral anti-infective therapy for this duration. However, the resistance profile of the confirmed XDR *K. pneumoniae* strain was not susceptible to oral antimicrobial suppression therapy, leaving a targeted oral therapy against *S. epidermidis* only. Therefore, it was decided to start an 3-month oral antimicrobial therapy with doxycycline (Figure 2).

Three months later, the patient was readmitted to our institution for the explantation of the drug-eluting cemented filler and another series of biopsies in order to prepare the left hip for arthroplasty (Figures 3C,D). The microbiological examination of the intraoperative biopsies confirmed successful eradication of *K. pneumoniae*, while *S. epidermidis* was still detected in extracted tissue samples of the left hip joint. Thus, the intravenous antimicrobial therapy with daptomycin was continued during the hospital stay and we decided to initiate an oral therapy with co-trimoxazole until hip arthroplasty (Figure 2).

**Figure 4:**
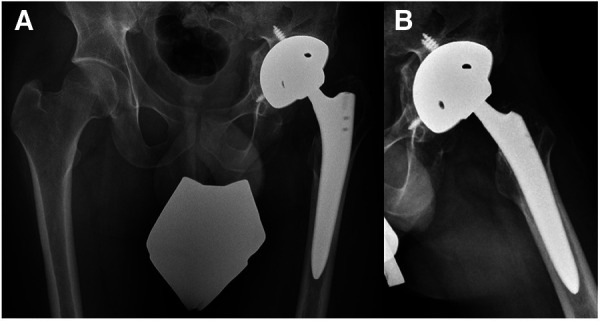
Pelvic x-rays (**A,B**) illustrating the postoperative result after hip arthroplasty implantation.

Another month later, the patient was readmitted to our institution. The patient presented with non-irritating skin conditions over the left hip joint, unremarkable laboratory results and a joint aspiration that showed no bacterial growth after 14 days of incubation. Thus, the primary hip arthroplasty, including intraoperative tissue sampling for microbiological analysis, was performed using a trabecular metal shell (TM modular multihole, Zimmer Biomet, Warsaw, Indiana) with an additional screw fixation in predetermined positions to ensure primary shell stability in combination with a meta-diaphyseally anchored hip stem (Smith + Nephew, Watford, UK) (Figure 4). After the surgical intervention the patient underwent regular clinical follow-up and an additional radiographic assessment 6 weeks and 3 month thereafter. The antimicrobial therapy was continued orally for 6 weeks with Rifampicin and Levofloxacin (Figure 2). Three month postoperatively, the patient presented pain free, walking without any assistive device. The 6 month clinical follow-up revealed implant survival without signs of periprosthetic joint infection.

## 3 Discussion

A shrapnel war injury shattering the hip joint and causing a secondary polymicrobial multidrug-resistant joint infection is a devasting condition in young patients. Furthermore, the management of FRI could be challenging due to bone defects as well as patient individual and environmental factors (23).

In this case report we reported a successful therapy of a polymicrobial FRI caused by extensively-drug resistant pathogens. Polymicrobial FRI occur in up to 30% of FRI cases overall, with Staph. epidermidis representing one of the most frequent pathogens and K. pneumoniae often associated with pelvic fracture, respectively (2). However, the extensive antimicrobial resistance of both pathogens in this case made the treatment even more challenging.

To achieve infection eradication, FRI therapy has to combine a sufficient surgical intervention with an appropriate antimicrobial therapy (1). Prompt surgical debridement in line with an intravenous antimicrobial therapy is crucial for infection control and the postoperative outcome (24, 25).

The duration of antimicrobial therapy in FRI is still controversial with common regimes between 6 and 12 weeks (26). Even when a recent study highlighted comparable outcomes between short-term and prolonged intravenous therapy, the presence of extensively drug-resistant pathogens in this case-report hindered early oral therapy (27). Therefore, intravenous therapy was continued in accordance to current treatment recommendations until microbial tissue sample remained negative (26).

Due to changes in pathogen species or acquired resistance, therapy adjustments are often necessary in such complex cases. Besides intravenous antimicrobial treatment, the local application of antibiotic-loaded bone cement has been shown to be beneficial for infection control (28, 29). In line with data by Morgenstern et al., who reported a significant reduction of infection rates due to local antibiotic application in open limb fractures, an antibiotic-loaded cemented filler was utilized to improve local antimicrobial therapy in our patient (30). Vancomycin, gentamicin and tobramycin are the most commonly added antibiotics in bone cement (31). Nevertheless, in some cases an individual composition is needed to address the infecting microbes. In this case report, an individualized antibiotic-loaded bone cement filler containing gentamicin, vancomycin and cefiderocol was used for local application and aided to successful infection control.

The successful cefiderocol application in bone cement has not been reported, yet. Even though this case illustrates the broad possibilities of cefiderocol application, its utilization as rescue drug in case of musculoskeletal infection is rarely studied. Only a small number of case series and case reports summarizes the cefiderocol mediated, successful treatment of infection due to multi-drug resistant gram-negative bacteria, and indicates its therapeutic potential to fight musculoskeletal infections (32–34).

Additionally, after repeated surgical interventions, CPP was used to support wound healing in this patient case. Recently, its feasibility in orthopaedic surgery has been reviewed and highlighted several promising applications (35). Particularly remarkable is CPPs effective action against biofilm and multidrug-resistent germs (36, 37).

After the successful infection eradication, the implantation of a total hip arthroplasty restored patients joint function and mobility. Displaced proximal femoral fractures are known to be at high risk of avascular necrosis resulting in an early hip arthritis (38, 39). Thus, THA has been shown a valuable treatment option to restore the joint function even in young patients, especially in the presence of a concomitant displaced acetabular fracture (40, 41).

In this case report a trabecular metal shell and a meta-diaphyseally anchored cementless hip stem was used. Nevertheless, there is still controversy in arthroplasty regarding implant design and mode of fixation, with advantages as well as disadvantages for cemented as well as cementless fixation (42, 43). The potential risk of greater loss of femoral bone stock when stem revision of a cemented stem become necessary could be an argument for using a cementless stem in our young patient, but the even higher risk of periprosthetic fracture in cementless stems has to keep in mind (43, 44). However, cementless femoral fixation has been shown to ensure a secure fixation and significantly improve the patient-reported outcome in young patients (45, 46). It has to be mentioned that not only diaphyseally anchored femoral stems have to be used in revision total hip arthroplasty. Recently, a small cohort study found good clinical outcome after revision total hip arthroplasty using a short femoral stem design (47).

Taken together, the objective of this case report was to highlight the crucial interplay between hip surgical intervention and long-term local as well as systemic antimicrobial therapy for the successful treatment of open FRI. Furthermore, we highlight the need for further research related to the treatment of multi-drug resistant musculoskeletal infections emphasizing the necessity to responsibly handle available rescue therapeutics.

However several limitations of this case description has to be noted. First of all, due to the uniqueness and restricted scope of a single case, there is limited generalizability. This case report focuses on a young patient without history of comorbidities. This could influence the patient-outcome significantly. Second, the infection-free implant survival is still a short-term observation and long-term data are needed to improve the strength of the described treatment strategy. Finally, the reported therapeutic approach is highly cost and recourse intensive and its implementation could only be feasible in specialized medical centers.

## 4 Summary

Overall, we report a successful total hip arthroplasty after a complex acetabular and femoral head war injury worsened by a difficult-to-treat polymicrobial joint infection. This case description highlight the importance of interdisciplinary collaboration in such complex cases as well as the excellent functional outcome properties of a staged hip arthroplasty intervention.

## Data Availability

The data analyzed in this study is subject to the following licenses/restrictions: The datasets generated and analyzed in the current study are not publicly available due to data protection regulations. Access to data is limited to the researchers who have obtained permission for data processing. Further inquiries can be made to the corresponding author. Requests to access these datasets should be directed to maximilian.fischer@med.uni-greifswald.de.

## References

[1] Moriarty TF Metsemakers W-J Morgenstern M Hofstee MI Vallejo Diaz A Cassat JE Fracture-related infection. Nat Rev Dis Primers. (2022) 8(1):67. 10.1038/s41572-022-00396-0 36266296

[2] Depypere M Sliepen J Onsea J Debaveye Y Govaert GA IJpma FF The microbiological etiology of fracture-related infection. Front Cell Infect Microbiol. (2022) 12:934485. 10.3389/fcimb.2022.934485 35873162 PMC9300981

[3] Metsemakers W-J Moriarty TF Morgenstern M Marais L Onsea J O'Toole RV The global burden of fracture-related infection: can we do better? Lancet Infect Dis. (2023):S1473-3099(23)00503-0. 10.1016/S1473-3099(23)00503-0 38042164

[4] Depypere M Morgenstern M Kuehl R Senneville E Moriarty T Obremskey W Pathogenesis and management of fracture-related infection. Clin Microbiol Infect. (2020) 26(5):572–8. 10.1016/j.cmi.2019.08.006 31446152

[5] Paryavi E Stall A Gupta R Scharfstein DO Castillo RC Zadnik M Predictive model for surgical site infection risk after surgery for high-energy lower-extremity fractures: development of the risk of infection in orthopedic trauma surgery score. J Trauma Acute Care Surg. (2013) 74(6):1521–7. 10.1097/TA.0b013e318292158d 23694882

[6] Horton SA Hoyt BW Zaidi SM Schloss MG Joshi M Carlini AR Risk factors for treatment failure of fracture-related infections. Injury. (2021) 52(6):1351–5. 10.1016/j.injury.2021.03.057 33863501

[7] Murray CK Obremskey WT Hsu JR Andersen RC Calhoun JH Clasper JC Prevention of infections associated with combat-related extremity injuries. J Trauma. (2011) 71(2):S235–57. 10.1097/TA.0b013e318227ac5f 21814090

[8] Lohr B Pfeifer Y Heudorf U Rangger C Norris DE Hunfeld K-P. High prevalence of multidrug-resistant bacteria in Libyan war casualties admitted to a tertiary care hospital, Germany. Microb Drug Resist. (2018) 24(5):578–84. 10.1089/mdr.2017.0141 29039717

[9] Pollak AN Ficke JR Injuries EW. Extremity war injuries: challenges in definitive reconstruction. J Am Acad Orthop Surg. (2008) 16(11):628–34. 10.5435/00124635-200811000-00003 18978285

[10] Miller AN Carroll EA Pilson HT-P. Transabdominal gunshot wounds of the hip and pelvis. J Am Acad Orthop Surg. (2013) 21(5):286–92. 10.5435/JAAOS-21-05-286 23637147

[11] Pazarci O Kilinc S Camurcu Y Bulut O. Total hip arthroplasty after hip joint gunshot injury. J Orthop Surg. (2019) 27(3):2309499019873113. 10.1177/2309499019873113 31496364

[12] Bell C Skibicki HE Post ZD Ong AC Ponzio DY. Gunshot wound resulting in femoral neck fracture treated with staged total hip arthroplasty. Arthroplast Today. (2022) 14:44–7. 10.1016/j.artd.2021.12.010 35242955 PMC8857266

[13] Salehi B Abu-Darwish M Tarawneh A Cabral C Gadetskaya A Salgueiro L Antimicrobial resistance collaborators global burden of bacterial antimicrobial resistance in 2019: a systematic analysis. Lancet. (2022) 399:629–55. 10.1016/S0140-6736(21)02724-0 35065702 PMC8841637

[14] Tan T Xu C Kuo F-C Ghanem E Higuera C Parvizi J. Risk factors for failure and optimal treatment of total joint arthroplasty for septic arthritis. J Arthroplasty. (2021) 36(3):892–6. 10.1016/j.arth.2020.09.020 33059964

[15] Blair JM Webber MA Baylay AJ Ogbolu DO Piddock LJ. Molecular mechanisms of antibiotic resistance. Nat Rev Microbiol. (2015) 13(1):42–51. 10.1038/nrmicro3380 25435309

[16] Haller S Kramer R Becker K Bohnert JA Eckmanns T Hans JB Extensively drug-resistant *Klebsiella pneumoniae* ST307 outbreak, north-eastern Germany, June to October 2019. Euro Surveill. (2019) 24(50). 10.2807/1560-7917.ES.2019.24.50.1900734 PMC6918589 31847948

[17] Eger E Heiden SE Becker K Rau A Geisenhainer K Idelevich EA Hypervirulent *Klebsiella pneumoniae* sequence type 420 with a chromosomally inserted virulence plasmid. Int J Mol Sci. (2021) 22(17). 10.3390/ijms22179196 PMC8431375 34502111

[18] Navon-Venezia S Kondratyeva K Carattoli A. *Klebsiella pneumoniae*: a major worldwide source and shuttle for antibiotic resistance. FEMS Microbiol Rev. (2017) 41(3):252–75. 10.1093/femsre/fux013 28521338

[19] Munoz-Price LS Poirel L Bonomo RA Schwaber MJ Daikos GL Cormican M Clinical epidemiology of the global expansion of *Klebsiella pneumoniae* carbapenemases. Lancet Infect Dis. (2013) 13(9):785–96. 10.1016/S1473-3099(13)70190-7 23969216 PMC4673667

[20] McCreary EK Heil EL Tamma PD. New perspectives on antimicrobial agents: cefiderocol. Antimicrob Agents Chemother. (2021) 65(8):e0217120. 10.1128/aac.02171-20 34031052 PMC8373209

[21] Izakovicova P Borens O Trampuz A. Periprosthetic joint infection: current concepts and outlook. EFORT Open Rev. (2019) 4(7):482–94. 10.1302/2058-5241.4.180092 31423332 PMC6667982

[22] Margaryan D Renz N Gwinner C Trampuz A. Septic arthritis of the native joint and after ligamentoplasty: diagnosis and treatment. Orthopäde. (2020) 49:660–8. 10.1007/s00132-020-03961-1 32737513

[23] Metsemakers W-J Onsea J Neutjens E Steffens E Schuermans A McNally M Prevention of fracture-related infection: a multidisciplinary care package. Int Orthop. (2017) 41:2457–69. 10.1007/s00264-017-3607-y 28831576

[24] Davis CM Zamora RA. Surgical options and approaches for septic arthritis of the native hip and knee joint. J Arthroplasty. (2020) 35(3):S14–8. 10.1016/j.arth.2019.10.062 32046824

[25] D’Angelo F Monestier L Zagra L. Active septic arthritis of the hip in adults: what’s new in the treatment? A systematic review. EFORT Open Rev. (2021) 6(3):164–72. 10.1302/2058-5241.6.200082 33841915 PMC8025707

[26] Metsemakers W-J Morgenstern M Senneville E Borens O Govaert GA Onsea J General treatment principles for fracture-related infection: recommendations from an international expert group. Arch Orthop Trauma Surg. (2020) 140:1013–27. 10.1007/s00402-019-03287-4 31659475 PMC7351827

[27] Li HK Rombach I Zambellas R Walker AS McNally MA Atkins BL Oral versus intravenous antibiotics for bone and joint infection. N Engl J Med. (2019) 380(5):425–36. 10.1056/NEJMoa1710926 30699315 PMC6522347

[28] Fleck EE Spangehl MJ Rapuri VR Beauchamp CP. An articulating antibiotic spacer controls infection and improves pain and function in a degenerative septic hip. Clin Orthop Relat Res. (2011) 469:3055–64. 10.1007/s11999-011-1903-1 21519937 PMC3183191

[29] Farhan-Alanie MM Burnand HG Whitehouse MR. The effect of antibiotic-loaded bone cement on risk of revision following hip and knee arthroplasty: a systematic review and meta-analysis. Bone Joint J. (2021) 103(1):7–15. 10.1302/0301-620X.103B1.BJJ-2020-0391.R1 33380204

[30] Fischer M Nonnenmacher L Möller A Hofer A Reichert J Matziolis G Psychological factors as risk contributors for poor hip function after periacetabular osteotomy. J Clin Med. (2023) 12(12):4008. 10.3390/jcm12124008 37373700 PMC10299103

[31] Berberich CE Josse J Laurent F Ferry T. Dual antibiotic loaded bone cement in patients at high infection risks in arthroplasty: rationale of use for prophylaxis and scientific evidence. World J Orthop. (2021) 12(3):119. 10.5312/wjo.v12.i3.119 33816139 PMC7995342

[32] Rose L Lai L Byrne D. Successful prolonged treatment of a carbapenem-resistant Acinetobacter baumannii hip infection with cefiderocol: a case report. Pharmacotherapy. (2022) 42(3):268–71. 10.1002/phar.2660 35075683

[33] Dagher M Ruffin F Marshall S Taracila M Bonomo RA Reilly R Case report: successful rescue therapy of extensively drug-resistant Acinetobacter baumannii osteomyelitis with cefiderocol. Open Forum Infectious Diseases. Oxford University Press US (2020). 10.1093/ofid/ofaa150 PMC7252278 32494581

[34] Zingg S Nicoletti GJ Kuster S Junker M Widmer A Egli A Cefiderocol for extensively drug-resistant gram-negative bacterial infections: real-world experience from a case series and review of the literature. Open Forum Infect Dis. (2020) 7(6):ofaa185. 10.1093/ofid/ofaa185 32548207 PMC7284008

[35] Nonnenmacher L Fischer M Haralambiev L Bekeschus S Schulze F Wassilew GI Orthopaedic applications of cold physical plasma. EFORT Open Rev. (2023) 8(6):409–23. 10.1530/EOR-22-0106 37289098 PMC10300842

[36] Daeschlein G Napp M von Podewils S Lutze S Emmert S Lange A In vitro susceptibility of multidrug resistant skin and wound pathogens against low temperature atmospheric pressure plasma jet (APPJ) and dielectric barrier discharge plasma (DBD). Plasma Processes Polym. (2014) 11(2):175–83. 10.1002/ppap.201300070

[37] Soler-Arango J Figoli C Muraca G Bosch A Brelles-Marino G. The Pseudomonas aeruginosa biofilm matrix and cells are drastically impacted by gas discharge plasma treatment: a comprehensive model explaining plasma-mediated biofilm eradication. PLoS One. (2019) 14(6):e0216817. 10.1371/journal.pone.0216817 31233528 PMC6590783

[38] Large TM Adams MR Loeffler BJ Gardner MJ. Posttraumatic avascular necrosis after proximal femur, proximal humerus, talar neck, and scaphoid fractures. J Am Acad Orthop Surg. (2019) 27(21):794–805. 10.5435/JAAOS-D-18-00225 31149969

[39] Konarski W Poboży T Śliwczyński A Kotela I Krakowiak J Hordowicz M Avascular necrosis of femoral head—overview and current state of the art. Int J Environ Res Public Health. (2022) 19(12):7348. 10.3390/ijerph19127348 35742595 PMC9223442

[40] Gee MJ Ajuied A Shah Z George M Bankes MJ. Systematic review of total hip arthroplasty in patients under 30 years old. Hip Int. (2013) 23(4):345–51. 10.5301/hipint.5000002 23475420

[41] Dunet B Tournier C Billaud A Lavoinne N Fabre T Durandeau A. Acetabular fracture: long-term follow-up and factors associated with secondary implantation of total hip arthroplasty. Orthop Traumatol Surg Res. (2013) 99(3):281–90. 10.1016/j.otsr.2012.12.018 23562708

[42] Murray D. Cemented femoral fixation: the North Atlantic divide. Bone Joint J. (2013) 95(11_Supple_A):51–2. 10.1302/0301-620X.95B11.32976 24187352

[43] Heckmann ND Chen XT Ballatori AM Ton A Shahrestani S Chung BC Cemented vs cementless femoral fixation for total hip arthroplasty after displaced femoral neck fracture: a nationwide analysis of short-term complications and readmission rates. J Arthroplasty. (2021) 36(11):3667–75.e4. 10.1016/j.arth.2021.06.029 34275708

[44] Holt G Hook S Hubble M. Revision total hip arthroplasty: the femoral side using cemented implants. Int Orthop. (2011) 35:267–73. 10.1007/s00264-010-1167-5 21165618 PMC3032104

[45] Chapot A Zambelli PY Merckaert SR. Functional and patient-related outcomes of total hip arthroplasty in patients younger than 20 years. Arthroplast Today. (2023) 20:101100. 10.1016/j.artd.2023.101100 36923059 PMC10009676

[46] Clohisy JC Oryhon JM Seyler TM Wells CW Liu SS Callaghan JJ Function and fixation of total hip arthroplasty in patients 25 years of age or younger. Clin Orthop Relat Res. (2010) 468:3207–13. 10.1007/s11999-010-1468-4 20668972 PMC2974894

[47] Mauch M Brecht H Clauss M Stoffel K. Use of short stems in revision total hip arthroplasty: a retrospective observational study of 31 patients. Medicina. (2023) 59(10):1822. 10.3390/medicina59101822 37893539 PMC10608113

